# The Saline Treatment of Dysentery

**Published:** 1900-02-17

**Authors:** 


### THE SALINE TREATMENT OF DYSENTERY.

Some time ago Major W. J. Buchanan, I.M.S., gave details of a series of 102 consecutive cases of dysentery treated by the administration of a saturated solution of sulphate of magnesium, with only one death. He now[1] returns to the subject and gives an account of 453 more cases of the same disease with only five deaths, which makes a total of 555 cases of dysentery treated by salines with only six deaths, or a percentage case mortality of only 1·08. The cases were of all types and forms, and are taken just as they came in from day to day. The average time in hospital was ten days for each case. On an average a relapse took place once in every 6·5 cases, and of the 453 cases 384 had only one attack each—a fact which of itself speaks for the efficiency of this line of treatment. In the first series of cases sulphate of magnesium was used, but in the last sulphate of sodium was the saline employed, one drachm in half an ounce of aqua fœniculi being given three or four times a day. The effect of this treatment is to produce a free but gentle purgation, and when bright yellow stools without a trace of blood or mucus are passed then the drug should be stopped, but resumed at once if blood or mucus should reappear in the stools. It is usually found that after five or six stools all blood and mucus have disappeared. In many cases they reappear in a day or so, and in such cases the drug must be given again. For chronic and relapsing cases the sulphate of sodium may be given for one or at most two doses, and then bismuth and soda, or other intestinal antiseptic, may be employed with diet; but for every exacerbation—that is, for every return of blood and mucus—a dose of sodium sulphate is given; or, if scybala are passed, a dose of castor oil and laudanum. Major Buchanan considers that, except in the limited doses mentioned above, salines are harmful where there is ulceration of the great intestine.

[1] Brit. Med. Jour., Feb. 10.

